# Our Voice NOLA: Leveraging a Community Engaged Citizen Science Method to Contextualize the New Orleans Food Environment

**DOI:** 10.3390/ijerph192214790

**Published:** 2022-11-10

**Authors:** Hasheemah Afaneh, Praveena K. Fernes, Emma C. Lewis, Abby C. King, Ann Banchoff, Jylana L. Sheats

**Affiliations:** 1 Health Sciences Center, School of Public Health, Louisiana State University, New Orleans, LA 70112, USA; 2 Department of Health Services Research and Policy, The London School of Hygiene and Tropical Medicine, London WC1H 9SH, UK; 3 Human Nutrition, Department of International Health, Johns Hopkins Bloomberg School of Public Health, Baltimore, MD 21205, USA; 4 Stanford Prevention Research Center, Stanford University School of Medicine, Stanford, CA 94305, USA; 5 Nutrition, Social Behavioral and Population Sciences, Tulane University School of Public Health and Tropical Medicine, New Orleans, LA 70112, USA

**Keywords:** citizen science, food access, digital health, food environment, social determinants of health

## Abstract

Objective: We employed the Our Voice citizen scientist method using a mobile application (app) to identify and contextualize neighborhood-level features influencing food access and wellbeing in New Orleans, Louisiana. Design: A three-phase, multi-method study comprised of: (1) a researcher-assisted tag-a-long neighborhood walk (referred to as a ‘journey’) with the Discovery Tool (DT) app to document neighborhood-level features via geo-coded photos and audio-recorded narratives; (2) a post-journey interview to enable citizen scientists to share their lived experiences; and (3) a community meeting with citizen scientists and local stakeholders. Setting: Various neighborhoods in New Orleans, Louisiana, USA. Participants: Citizen Scientists (i.e., residents) aged 18 years and older. Main Outcome Measure(s): Features that influence food access and health behaviors. Analysis: Descriptive statistics and a thematic content analysis were conducted to assess survey and app data. Results: Citizen scientists (N = 14) captured 178 photos and 184 audio narratives. Eight major themes were identified: safety; walkability; aesthetics; amenities; food; health services; neighborhood changes; and infrastructure/city planning. The post-journey interview provided insights around the abovementioned themes. The community meeting demonstrated the willingness of citizen scientists and stakeholders to convene and discuss issues and relevant solutions. Conclusions and Implications: Findings demonstrate the ability of technology and citizen science to help better understand the complexities of New Orleans’ past, present and distinct culture—and implications for food access and wellbeing in the context of trauma in an urban ecosystem.

## 1. Introduction

Social determinants of health (SDOH) are encompassed in the neighborhoods that we live in, the parks that we play in, the cities that we work in, and the communities that we pray in [1,2]. They subsequently impact and shape our lives—and our health—as we navigate social and built environments [1]. The social environment is made up of factors such as social support, social networks, social deprivation, income inequality, racial discrimination, social cohesion, and social capital within neighborhoods [3]. The built environment, meanwhile, is characterized by human-made structures and spaces, such as buildings, roads, bridges, parks, transportation systems, and houses [4,5]. According to Matthews and Yang [6], “both the social and built environments are expected to be associated with variations in individual health across neighborhoods (p. 171)”. Thus, within and across these environments, there are factors that support or hinder the ability to engage in healthy lifestyle behaviors, and subsequently, impact health outcomes [1,7,8]. The built environment is an established contributor to disparities with respect to the availability of, and access to, opportunities to engage in key behaviors for optimal health and wellbeing (i.e., physical activity, healthy eating). For example, living in an environment that lacks green space and/or healthy food options may make it difficult to engage in related health behaviors. This may also have implications for the extent to which individuals are exposed to, and engage with, their local neighborhood and surrounding communities [9].

Often praised for rich social and food cultures, the people and city of New Orleans, Louisiana have demonstrated resilience over the last 300+ years. However, there are clear, long-standing health disparities, inequalities, and inequities among racial/ethnic minority and vulnerable populations. Across the landscape of the city’s social and built environments, these disparities, inequalities, and inequities bring SDOH to life in ways that require thoughtful examination and problem-solving. The simultaneous consideration of SDOH within the political context of the shocks and stresses suffered by the city and people of New Orleans in recent history is equally important [10,11]. The large-scale events of (1) Hurricane Katrina in 2005; (2) the Great Recession in 2009; and (3) the Deepwater Horizon Oil Spill in 2010 greatly exacerbated health disparities within the city [12]. These shocks, coupled with deeper systemic stresses—particularly those related to disparities and structural power relations—have resulted in the New Orleans community being particularly vulnerable to a range of health risks and less than optimal health outcomes [13,14]. For example, among Black residents in two New Orleans zip codes within five miles of one another, there was a 25-year difference in life expectancy in 2018 [15]. The abovementioned reference to disparities goes beyond physical health. Food access and availability, as well as food security, are persistent challenges faced by New Orleans residents. Food access is one’s relative accessibility to sources of food, which may be impacted by individual- (i.e., income, transportation) and neighborhood- level (crime, availability of public transportation) factors [16]. Nearly one-quarter of New Orleans households fall below the federal poverty line, and 19% do not have a means of transportation [17,18]. Recent data show that one in four New Orleans residents are food insecure (i.e., a lack of availability and access to safe, nutrient-dense foods) [17,19]. Black residents in particular, have been found to reside in neighborhoods considered food deserts (i.e., inadequate levels of access to retail outlets selling healthy and affordable foods), more so than their non-Black counterparts [11]. According to Rose and O’ Malley [13], disparities in food access increased after Hurricane Katrina. Most grocery stores are located >1 mile away from the average New Orleans household [17,20]. Further, post-Hurricane Katrina (also referred to as ‘the storm’) supermarket access declined from 31 supermarkets per census-tract neighborhood pre-Katrina, to only 15 supermarkets per census-tract neighborhood post-Katrina [11]. For example, 15 years post-Katrina in 2020, there was only one grocery store located in the Lower Ninth Ward. This community is predominantly Black (92.9%) and was the most affected geography by Hurricane Katrina. Opened by a community resident and advocate in 2014, the modest Lower Ninth Ward Market is an asset to residents and provides services that reflect the needs of the community (e.g., the food store is also a barber shop, self-serve laundromat, computer center, and walk-up restaurant) [17,21]. 

In addition to the Supplemental Nutrition Assistance Program (SNAP), Special Supplemental Nutrition Program for Women, Infants, and Children (WIC), and other federal resources, there has been a surge in novel efforts to address food access, food availability and food security—many of which are connected to the food system [13]. The New Orleans food environment is diverse in that there are multi-sector collectives, organizations, and creative social innovation efforts throughout the city [13]. Downs and colleagues [22] recently expanded the definition of the food environment to “the consumer interface with the food system that encompasses the availability, affordability, convenience, and desirability of foods and beverages in wild, cultivated, and built spaces that are influenced by the socio-cultural and political environments within which they are embedded (p. 5)”. While diverse, the blueprint for present-day New Orleans has been largely shaped by the city’s original redlining maps from the early 1900s, which identified sections of the city deemed to be poor financial risks. These maps had a lasting impact on the housing market and zoning areas, and continue to affect the food environment with regard to determining where community grocers, restaurants, and other businesses can operate [23]. Early redlining also led to the denial of credit and housing to low-income, racial/ethnic minority families [23]. Subsequently, present-day redlined neighborhoods are also the same areas where there is a documented disproportionate exposure to social and physical disorder and where residents experience challenges such as food access, neighborhood blight, poor quality housing, lack of services, and vacant lots [24]. Amid these factors, there is an undeniable ‘sense of place’ felt by many New Orleanians. As described by Chamlee-Wright and Storr [25]. when referring to Lower Ninth Ward residents returning after Hurricane Katrina, “Returning residents believe[d] that New Orleans in general (and their Ninth Ward neighborhoods in particular) possess[ed] a unique bundle of characteristics that, when taken together, [could not] be found or replicated elsewhere (p. 615)”.

The *Neighborhood and Built Environment* and the *Social and Community Context* are two of five key SDOH where multi-sector, multidisciplinary work should be conducted to address problems facing individuals and communities [1,2]. The present study, titled *Our Voice NOLA* (New Orleans LouisiAna), leveraged the use of the *Our Voice* Citizen Science method developed by the Healthy Aging Research and Technology Solutions lab at the Stanford Prevention Research Center, Stanford School of Medicine [26]. *Our Voice*, which has been used previously to assess food environments, is a community-based participatory research (CBPR) method that merges photovoice techniques with citizen science to facilitate advocacy around community-level social, economic, and political determinants of health.

Often, social science research, like ethnography, relies heavily on spoken and written interviews that typically require individuals to recall neighborhood experiences while away from the neighborhood context [26]. There is also a tendency for social epidemiologists to squeeze the social determinants of health into covariates in a regression model [27], p. 5. Quantifying social experiences in this way often misses factors that contribute to understanding the full “story” (e.g., historical and social contexts, networks, and subjective understandings of place) [27], p. 6. Spatial quantitative measures typically used in the field yield rigorous objective environmental data—but may fail to capture residents’ “Contextual intelligence” and subjective experiences in their environments [27].

*Our Voice* meets these challenges by capturing the economic, social, cultural, political concepts around health issues through open-ended walks with citizen scientists; spatial qualitative methods provide in-depth subjective information contextualized in situ. In the spirit of the idiom, ‘a picture is worth a thousand words’, photographs taken by citizen scientists prompt broad and deep reflection and dialogue. Collecting narrative-laden data on-the-move not only offers a more dynamic account than stationary locales, but it also has the potential to highlight the environments of citizen scientists as ‘actors’ in practices associated with health, as demonstrated in previous studies on the *Our Voice* method [28,29].

In the present context, the *Our Voice* method was used to: (1) document citizen scientists’ (i.e., community residents) experiences navigating their social, built, and food environments in the context of their neighborhood; (2) identify factors that promote and/or hinder the ability to engage in healthy lifestyle behaviors, with an emphasis on food-related behaviors; and (3) facilitate a community-engaged process with citizen scientists, members of the community-at-large, and key local and state stakeholders to discuss the most salient community concerns and potential solutions. The *Our Voice* method is novel in that it combines the above-mentioned participatory methods with rigorous citizen science data collection methods and mobile technology (i.e., Healthy Neighborhood Discovery Tool [DT] app), which enables the collection of geo-tagged photos and audio narratives by citizen scientists.

## 2. Materials and Methods

### 2.1. Participants and Recruitment

The study protocol and methods were approved by the Tulane University Institutional Review Board. As part of the study protocol, citizen scientists were recruited from Summer 2018 to Winter 2018 using flyers posted in various community and public spaces, such as faith-based organizations, food pantries, and community health centers. Eligibility criteria included being: (1) aged 18 years or older; (2) a resident of New Orleans; and (3) competent in the English language. Citizen scientists were directed to call a telephone number if they were interested in participating. Once eligibility was confirmed through a telephone screening, the research team scheduled an appointment to have each citizen scientist meet with one to three members of the research team in a comfortable location in their community. In the context of this paper, ‘community’ was defined as one’s neighborhood of residence, or the place where they spent most of their time. Informed consent was obtained from all subjects involved in the study. Upon meeting, the research team reviewed the study consent form with participants to describe the study and what it entailed. They then asked them to state in their own words their understanding of the study and what was being asked of them. Once complete, researchers obtained verbal consent. Once consent was obtained, the research team proceeded with administering the study survey.

### 2.2. Instrument

The study survey was quantitative and included demographic items (i.e., gender, age, health status) as well as select items from validated surveys on the topics of food security [30], perceived neighborhood health [31] and the availability and quality of food [32]. Drafts of the survey were reviewed by staff from our partner, Second Harvest Food Bank of Greater New Orleans and Acadiana. Subsequent feedback was incorporated into the final version.

### 2.3. Procedures

#### 2.3.1. Neighborhood Journey

Citizen scientists were asked to complete the study survey. If they had difficulty, a member of the research team offered to administer the survey verbally. Once complete, they were introduced and briefly trained (≤5 min) to use the Discovery Tool (DT) mobile application (app). The easy-to-use app enables residents to identify and document neighborhood features by capturing geocoded photographs, audio narratives, and GPS-tracked walking routes—with a full description provided elsewhere [26,29]. Using the DT, a tag-a-long ‘journey’, or walk, ensued with one to three members of the research team being led by the citizen scientist through their community. During the journey, each citizen scientist captured geocoded photos with accompanying audio narratives (1:1 match) of neighborhood features that they perceived to have an influence on their ability to access food and on their overall health and wellbeing. Through the app, citizen scientists indicated a general rating for photos as good, bad, or neutral with respect to their health or wellbeing, which aided the researchers, and subsequently, the citizen scientists at the community meeting, to analyze facilitators and barriers to healthy living in New Orleans neighborhoods. Although the research team was present, their participation during this phase was limited to providing technical support and/or answering study-related questions.

#### 2.3.2. Post-Journey Interview

Following the journey, a brief, reflective, semi-structured oral interview was conducted. This phase was an additional component to the *Our Voice* method that, at the time of implementation, was unique to *Our Voice NOLA* [26,29,33]. Specifically, *Our Voice* studies typically have a data review meeting [33,34], whereas the current study provided an opportunity for residents to reflect immediately after their journey. The aim of this new element to the methodology was to allow citizen scientists to share their experience of the journey—framed as a ‘story’—to deepen the experience and enable them to reflect on and analyze their observations. Encapsulating citizen scientist’s life stories into static images and a brief audio caption may flatten more complex experiences. The post-journey interview provided a means to test a new stage in the methodology that delves into deeper discussion regarding citizen scientist’s interactions with their environments. Thus, throughout this process, citizen scientists were encouraged to be mindful and intentional while explaining their lived experiences to the research team members. Four questions were asked: (1) “Why did you choose to participate in this study?”; (2) “What made you want to share your story?”; (3) “How did going on this walk make you think about your neighborhood?”; and (4) “Has telling your story allowed you to see what strengths and resources you do have (i.e., how has this process helped you see what you may have not otherwise recognized without sharing it as a story?)”. Upon completing the interview, citizen scientists were given a tote bag containing healthy snacks provided by Second Harvest Food Bank of Greater New Orleans and Acadiana.

#### 2.3.3. Community Meeting

The final phase was a researcher-facilitated community meeting for the citizen scientists, the community-at-large, and key stakeholders from relevant community organizations (Table 1), with the goal of presenting the *Our Voice NOLA* methodology and study findings and identifying themes around key issues and relevant solutions. Citizen scientists received phone calls with reminders about the event and asked to share with their networks; emails along with personal phone calls were made to community organizations; and flyers were posted in the community to promote attendance. Community meeting participants viewed citizen scientists’ photos and audio narratives in a museum gallery exhibition style format, with photos being selected for inclusion based on the following: (1) the clarity of the photo; and (2) the relative impact of the accompanying narrative. Open and guided discussions among all meeting participants were conducted to identify themes of key issues and relevant solutions. Copious notes were taken by the research team during this time.

### 2.4. Data Analysis

Descriptive statistics for survey data were analyzed using SPSS Statistics Version 27.0 (IBM, Chicago, IL, USA). The research team used an inductive approach within the scope of thematic analysis, which is best described as, “a method for identifying, analyzing, and reporting patterns (themes) within data” [35], p. 79. This analysis included the following: (1) downloading and transcribing the photos and audio-recordings; (2) independently providing an initial review of the raw data by four of the research team members; (3) meeting to discuss similarities and differences observed in data coding; and (4) generating a coding scheme based on these discussions until consensus was reached. Interview recordings were downloaded to a secure server and transcribed by the research team. Taking an iterative and systematic approach, the research team applied principles of grounded theory [36] to analyze the interview data, which included: (1) line-by-line open coding (2) consolidating into focused codes via consultation with team members and memoing (documenting reflections and learnings from the data); and (3) integrating diagrams to inform analytic interpretation and theme development [37,38].

## 3. Results

### 3.1. Sample and Community Description

There were 14 citizen scientists (N = 14) who participated in this research. The sample size is in range with other *Our Voice* research producing valuable insights [26,34]. Citizen scientists were aged between 23 to 75 years with the majority being female (n = 12) and African American (n = 11). Most citizen scientists were New Orleans natives (n = 9) and half (n = 7, 50%), reported living in New Orleans during Hurricane Katrina. Nearly three-quarters perceived their health to be ‘good’ (n = 10). One citizen scientist used an assistive device during study activities (i.e., scooter). Journeys were conducted in seven New Orleans neighborhoods: Hollygrove, Bayou St. John, Broadmoor, Treme-Lafitte, Uptown and the Lower Garden District. Among the fourteen citizen scientists, twelve journeys were implemented in communities in which they lived; whereas two were implemented in communities in which they worked. Almost two-thirds (64%, n = 9) reported being members of their community for 5–10 years. With regard to food security, almost half (42%, n = 6) reported being hungry in the last 12 months because there was not enough money for food; and 36% (n = 5) reported participating in supplemental food programs or visiting food pantries. Twenty-nine percent (n = 4) reported that food stores (grocery stores) were generally not within walking distance to their home or work; and 64% (n = 9), perceived the fruits and vegetables that were available within or outside of walking distance in their community to be of poor quality. When asked to think about their community as a place to live (as opposed to work), 66% (n = 8) reported ‘agree’ or ‘strongly agree’ to violence being a problem; and 83% (n = 10) reported ‘agree’ or ‘strongly agree’ to people in their community being willing to help their neighbors. Among all citizen scientists—whether living or working (n = 14) in their respective community—64% (n = 9) rated their neighborhood overall as ‘good’ or ‘very good’.

### 3.2. Discovery Tool (DT) Journey Data

A total of 184 audio narratives and 178 photographs were captured by citizen scientists. Due to technical difficulties, five photos were lost. A secondary back-up mobile phone for the study was used to capture photos and audio narratives on journeys (i.e., walks) to prevent further data loss. Geo-tagged data were not available from the back-up resource due to technical issues with the DT app, and therefore, geo-spatial aspects of the photos were unavailable for analysis. When the research team reviewed the photos (n = 178), they identified eight broad themes, as follows: (1) safety; (2) walkability; (3) aesthetics; (4) amenities; (5) food; (6) health services; (7) neighborhood changes; and (8) infrastructure/city planning (Table 2). For each theme, both facilitators and barriers were often present in each photo. For example, one citizen scientist rated the availability of a local food store in their neighborhood positively; but also rated it negatively because there were minimal fresh foods and an abundance of processed foods. Another citizen scientist positively rated the presence of sidewalks in her neighborhood, given the dearth of sidewalks on neighboring streets. However, they also captured a car parked on the sidewalk, noting that it forced pedestrians to walk in the street and thus rated it negatively as well. Further, the theme ‘neighborhood changes’ was perceived to be positive by a citizen scientist for its representation of necessary growth; but these changes were also perceived to be negative by others for their representation of gentrification and unwanted changes to the neighborhood’s culture. The above examples reflect the duality of neighborhood elements.

### 3.3. Post-Journey Interview Data

The research team identified three categories of themes from the post-journey interview data, including: (1) neighborhood context (neighborhood changes and variation, community membership, post-Katrina sense of place, neighborhood sense of pride); (2) reasons for participating (individual and community level); and (3) realizations during the process (realized strengths/positives, realized problems/negatives, normalization of health behaviors, ability to advocate). Definitions, sub-categories, and exemplary quotes from citizen scientists are displayed in Table 3 for each category. With respect to neighborhood context, Hurricane Katrina and gentrification (Neighborhood Changes) were top-of-mind for citizen scientists. Although the storm occurred 15 years ago, Hurricane Katrina has had lasting implications for each of the identified present-day thematic issues in Table 2 (i.e., safety, walkability, aesthetics, amenities, food, health services, neighborhood changes and infrastructure/city planning). While some citizen scientists perceived neighborhood changes to be representative of ‘growth’, other citizen scientists referred to the ‘growth’ as ‘gentrification’. They felt that gentrification, as well as subletting homes as ‘vacation rentals’, decreased neighborhood safety and neighborhood cohesion and diluted the culture of the city and neighborhood, respectively. Yet, regardless of citizen scientists’ length of residence and perceptions about neighborhood context (e.g., there was an overall sense of pride and desire for neighborhood cohesion. This finding aligns with the community-level rationale that citizen scientists provided for participating in *Our Voice NOLA*, which was generally to be a voice for the community, to obtain improvements, and to inform future decisions. Several realizations elicited by implementing the *Our Voice* method emerged during the post-journey interview as well. Specifically, using the DT enabled citizen scientists to observe, acknowledge, document and reflect on community strengths and problems, as well as the normalization of health barriers to which they may have been previously desensitized to. For example, not having sidewalks, having a sidewalk that ended unexpectedly, or finding obstructions located in the middle of a sidewalk had been normalized to most. It was through the DT process that citizen scientists realized that they had adapted to such situations by automatically moving into the street to walk. Additionally, some citizen scientists took a moment to look at, or ask questions about, the Blue Bikes populated throughout the city for the first time. Blue Bikes refers to a city-wide bike-sharing program with a nominal annual subscription rate for Supplemental Nutrition Assistance Program enrollees [39]. Lastly, overall, citizen scientists understood and expressed the need for and importance of advocating for themselves and their community. However, when it came to knowing *who* to discuss issues with—or where the resources were—their responses were less clear.

### 3.4. Community Meeting

A two-hour researcher-facilitated community meeting was held in December 2018. While all citizen scientists were invited to attend and received follow-up reminder calls, 7 attended. shared ride service was arranged for those without transport. Members of the community-at-large (n = 5) and stakeholders representing organizations throughout New Orleans (n = 14) (Table 1) also attended amid the diversity of promotional efforts. Meeting participants were presented with the *Our Voice NOLA* methodology and study findings by members of the research team. This also included viewing and listening to a selection of the captured photos with their accompanying audio narratives, as well as deidentified insights from the post-journey interviews. Afterward, meeting participants took 30 min to view the citizen scientists’ photos and accompanying narratives (i.e., full or partial quotes from audio recordings that captured the essence of what was conveyed during the journey), which were displayed in a museum gallery exhibit format. At the end of the ‘gallery walk’, citizen scientists elaborated on their experiences with their peers, who commented and shared their own experiences.

Community members-at-large in attendance shared similar issues represented in the *Our Voice NOLA* data, such as a lack of sidewalks, limited grocery store access, vacant lots, and excessive blight, all of which facilitated a lively dialogue. They made note of certain areas in the city that were damaged during Hurricane Katrina and a subsequent flood at least a decade later, emphasizing that these areas remain untouched today (i.e., the Ninth Ward and New Orleans East). Citizen scientists and community members-at-large were asked to collectively think about what they heard and viewed during the presentation and ‘gallery walk’; and identify key themes that they felt were represented across the *Our Voice NOLA* data. They had not been provided with the researcher-identified themes of key issues beforehand. Once everyone reconvened, community members-at-large and citizen scientists voiced what they perceived to be themes representing key issues from the data. Once presented, the researcher-identified themes were revealed side-by-side for comparison. While the terminology used may have been different among the community residents relative to the researchers (i.e., ‘access to healthcare’ vs. ‘health services’; ‘food’ vs. ‘no grocery stores’), there was a one-to-one theme match, with additional themes such as ‘the forgotten Ninth Ward,’ ‘lack of services’ (generally speaking) and ‘water quality’ being generated by community residents.

Both citizen scientists and their peers were aware of the problems that existed in their respective neighborhoods, and the city overall. They expressed that although they came from different neighborhoods, they were actively seeking solutions to address these problems and shared a common desire for change. At the end of the community meeting, stakeholders spoke about their respective organizations’ missions, available services, and upcoming events applicable to the key themes and other issues revealed during the meeting. Proposed next steps included: (1) continuing this line of research across the distinct New Orleans neighborhoods to obtain more community perspectives; (2) sharing programs, services, and resources with meeting attendees; and (3) making the results available on a public website with accompanying resources.

## 4. Discussion

The *Our Voice* method is a digital approach that enables community entry, and individual-to-group ‘consultation’ with citizen scientists, the community-at-large, and stakeholders, at different time points and levels of engagement. This study leveraged the DT in urban New Orleans and provided valuable insights that illuminated the cross-sector and multi-disciplinary problems to be solved. Employing *Our Voice* allowed for a focus beyond individual level change, while simultaneously stressing the importance of examining and gaining perspective at all levels of analysis.

This method also speaks to [40] argument that “a more relational or integrated approach to defining urban places and acting on health equity can complement other approaches and improve the ability of public health to meet 21st century challenges (p. 1)”. Citizen scientists often brought up elements of the environment that were simultaneously social, built, natural, human, and non-human. For instance, participants narrated how Hurricane Katrina (arguably a human-made ‘natural disaster’) affected the metabolism of the city, from damaged infrastructure to increased stress and trauma. These examples problematize environmental and public health scholars’ tendency to frame places as either ‘natural’ or ‘built’ [40]. While citizen scientists and community members identified influential factors and experiences in the neighborhood environment within the context of Hurricane Katrina, the only related survey item was whether or not they were living in New Orleans during Hurricane Katrina. In the book *Stories of Survival (and Beyond),* Writes [41] likens the trauma of Hurricane Katrina to “…seismic tremors that persist long after an earthquake has passed”, and suggests that “If you don’t feel it yet, listen to the voices of some of the people that do”.

Through this research, the voices of citizen scientists conveyed neighborhood factors that influenced food access and overall health and wellbeing. There was an a priori intention to focus primarily on food access, yet what this approach revealed was that other distinct—and inter-related, influential factors can and did emerge. This finding demonstrates the complex, overlapping nature of social determinants of health [42]—which was also found in previous *Our Voice* studies exploring food access and availability [34]. Both the photos and audio narratives revealed thematic issues (Table 2; i.e., walkability, food access, availability of health services, amenities) that were primarily—with the appropriate allocation of resources—*modifiable*. The subsequent post-journey interview often took place at a citizen scientists’ kitchen table, on their porch, or in a public sitting space. The informality of these spaces may have led to increased trust and more meaningful discussion, which in turn led to greater insights. Interview data provided a lens through which to view and analyze data captured from the survey and DT.

Comparing the themes developed by researchers with those developed by citizen scientists and community members-at-large demonstrates the value of their analysis and embodies CBPR’s emphasis on participation of community members as co-researchers and the role of the researcher as a co-learner [43]. This exercise also added richness to the researchers’ understanding of the identified themes based on the lived experiences of the community residents present at the meeting—and the perspective of stakeholders with resources, programs, and services available to potentially address issues within and across the themes. Perceived inequities was a central component in all of the themes, as prefaced by the meeting participants. For example, ‘the forgotten Ninth Ward’ was a major theme that meeting participants felt was exemplified in the DT data. ‘The forgotten Ninth Ward’ theme, for example, spoke to disinvestment (e.g., no supermarket) and social determinants of health, in the Ninth Ward specifically, but this sentiment could potentially apply to multiple neighborhoods across the city, as the need for adequate food stores, services, and repair was persistent overall. The community meeting illuminated the willingness of citizen scientists, community members-at-large, and stakeholders to hear from one another, discuss, and advocate for issues; and collectively identify solutions to address them.

While *Our Voice* works towards addressing some of the political determinants of health [26] by documenting issues such as the ‘forgotten Ninth Ward’ and Hurricane Katrina—and optimizing the power of spatial qualitative methods, the method could benefit from better connecting large-scale political, social and economic processes to local health and well-being [44]. In this context, the DT resembled ‘counter-mapping’, a social process whereby community members make their own meaningful ‘maps’, or delineated pathways through their neighborhoods, as alternatives to those used by discriminatory top-down processes such as redlining [45,46]. Despite several examples that cited local power dynamics and politics, *Our Voice* research that speaks to wider structures of oppression and injustice is an emerging area for the methodology [47]. While *Our Voice* captures the current material manifestations of social-natural-built environments, the method risks taking a cursory glance at the historical and structural context of a community’s struggle. This brings up a limitation in CBPR regarding scale: by honing in on the micro-local approach, macro-democratic processes may be neglected [48]. Additionally, it brings up a strength of the method: in a departure from focusing on abstract, macrostructural forces like capitalism and the state that can make change seem impossible, this method opens up ways to locate dynamic social relations, power and resistance from within the micropolitics of activities and interactions themselves [49].

## 5. Conclusions

The complex nature of New Orleans’ social, built, and food environments, coupled with past and continued present-day shocks, stresses, inequities, and racial and health disparities, warrants further examination. *Our Voice NOLA* findings contribute to and extend the *Our Voice* evidence base [26] as well as digital health, citizen science, and food access research; and efforts to investigate SDOH in vulnerable, yet resilient, communities such as New Orleans. While seemingly paradoxical, evidence has shown that vulnerability and resilience can co-existence particularly urban ecosystems that have experienced traumas and challenges involving social and ecological disruption and devastation, such as Hurricane Katrina [12]. Usamah and colleagues [50] refer to community members’ resilience in relation to severe weather events, economic limitations, inadequate housing, and beyond as ‘*strength in the presence of stress*’. They suggest that affected communities have inbuilt resilience due to the perception that “disasters as part of life”, and facilitate social connectedness.

With regard to implications for practice, the application of this method may provide a blueprint on which future work in New Orleans can be built. Similar to previous research exploring perceptions around health within the context of digital innovations in New Orleans, the long-lasting implications of Hurricane Katrina and the city’s unique culture were salient issues for citizen scientists [51]. These issues collectively framed the interpretation of data obtained during the journey; storytelling during the post-journey interview; and discussions during the community meeting. According to Writes [41] while Hurricane Katrina was a severely stressful, traumatic, and challenging event, early on the storm was perceived by some to be a catalyst for change, which instigated new commitments to the city. There are vested individuals and entities, as demonstrated through this work, who want to see change across the city and have no doubts regarding its survival. For example, nearly 10 years post-Katrina, the Rockefeller Foundation [52] applauded New Orleans’ resilience in the face of adversity and recognized investments and opportunities—stating that the city is “living, breathing, thriving proof that cities could meet those challenges head on, and, with a bit of ingenuity, and a willingness to acknowledge and learn from past mistakes, even bounce back stronger from them”. Findings from this study indicate that while citizen scientists generally reported low food access (i.e., food stores, quality fruits and vegetables) perceived that violence was a problem, they felt that people living in their community were willing to help their neighbors and rated their neighborhoods as a ‘good’ or ‘very good’ place to live and/or work (relative to one-third rating it as ‘bad’ or ‘very bad’). This may speak to Usamah and colleagues’ [50] previously mentioned notion of ‘strength in the presence of stress,’ as well as the culture and collective pride of New Orleanians. As expressed in *Stories of Survival (and Beyond)* [41], the strength of the New Orleans people and culture will enable it to survive and be resilient because they—and the city, have always had to.

In closing, *Our Voice NOLA* has provided valuable insights around the lasting implications of a natural disaster and may present strategies that can be used to better understand the implications of more recent ‘traumas,’ such as the COVID-19 pandemic and Hurricane Ida, through equity and social-ecological lenses. This work has important implications for New Orleans residents, community leaders, and local organizations as they work towards improving the intertwined social, built, and food environments. Previous Our Voice studies have demonstrated the inherent value of the methodology for advocacy and change [26,28,29,33,34] amongst researchers, public health practitioners, community organizations and others. The current study demonstrates the feasibility of using the DT identify issues; and convene residents and other key stakeholders to begin initial conversations on: problem identification and their root causes; existing and/or new solutions and changes that need to take place; and insights that may inform which inter- and multi-sector collaborators might play a role in facilitating and implementing said changes. A major strength of the present study is the utilization of multiple methods, including quantitative, qualitative, and technology-based instruments, to acquire an understanding of factors impacting food access and wellbeing. Practitioners of related participatory models can consider incorporating *Our Voice* best practices when developing other projects that emphasize community-driven data gathering and solution generation.

This study also has limitations that should be acknowledged and addressed. One limitation was the small sample size. However, this sample size was in range with other *Our Voice* research, which has shown that consensus in a community locale can typically be reached with as few as 8–10 citizen scientists [26,33,53], *Our Voice* research has demonstrated that insights typically reach a point of consensus with such sample sizes—and thus, larger sample sizes may not be necessary in many cases [26]. Yet, while the community meeting yielded five community members (seven scientists, 14 community organizations), having greater community representation would have contributed to further and deeper insights. Convening community meeting attendees (i.e., citizen scientists and members of the community-at-large) and their validating and confirming the researcher-identified themes from data collected by the 14 citizen scientists in the presence of key stakeholders was promising with regard to using the tool within the context of New Orleans. The process of the present study—the surveying, the journey, and the post-journey debriefing—was time-consuming, and this could have impacted what was shared. Finally, lack of funding is a limitation for recruitment. It is our hope that the value of digital approaches to community-engaged citizen science research is reflected in future funding streams.

## Figures and Tables

**Table 1:** Community Organizations in Attendance at the Community Meeting.

Organization	Domain	Website
TopBox Foods	Food access	https://www.topboxfoods.com/new-orleans/home
Ready Responders	Medical care access	https://readyresponders.com/
Louisiana Budget Project	Hunger and poverty reform	https://www.labudget.org/
NOLA Children’s Health Project	Pediatric care access	https://communitypediatrics.tulane.edu/
Bike Easy	Affordable transportation	https://bikeeasy.org/
American Heart Association	Cardiovascular health and advocacy	https://www.heart.org/
GirlTrek	Black women’s health	https://www.girltrek.org/
Sankofa	Food access	https://sankofanola.org/
Culinaria Center	Food system policy reform	http://gopropeller.org/ventures/culinaria-center-for-food-law-policy-and-culture/
FitNOLA	Nutrition and physical activity	https://www.nola.gov/health-department/fit-nola/
Louisiana Office of Public Health	Government entity to improve health	https://ldh.la.gov/index.cfm/subhome/16
Oko Vue Produce Co.	Food production	https://www.makingroceriesmarket.com/
ReFresh Project	Fresh food hub	http://broadcommunityconnections.org/projects/refresh
New Orleans Health Department	Government entity with the mission to protect, promote and improve health	https://www.nola.gov/health-department/

**Table 2:** Major Themes Identified in Citizen Scientists’ Photos and Audio Narratives.

Theme	Definitions	Exemplary Quotes
1. Safety	Perceived feeling of being unlikely to experience danger, risk, or injury	Violent crimes, Petty theft, Knowing Neighborhoods, “Vacation rentals” bringing strangers into the neighborhood
2. Walkability	Perceived ease of getting around the neighborhood on foot	Broken sidewalks, lack of sidewalks
3. Aesthetics	Perception of how pleasing the neighborhood is at face-value	Excessive potholes, Blight, Abandoned houses
4. Amenities	Perception of services available within walking distance that serve a purpose for the neighborhood residents	Gym, Library, Basketball court, Restaurants
5. Food	Perception of the places that serve or sell any food products within walking distance and appeal to neighborhood residents	Proximity, Access, Availability, Affordability, Quality
6. Health Services	Perception of the places within walking distance that provide health-related services to neighborhood residents	Availability, Accessibility, Urgent care, Community clinic
7. Neighborhood Changes	Perceived direct or indirect outcomes of transitions in the neighborhood that have been identified by residents	Growth, Gentrification, Diversity, Blue bikes, Greenway
8. Infrastructure/City Planning	Perceived physical or social objects or services that have been put in place in the neighborhood by the city of New Orleans	Sidewalks, Potholes, Bike lanes

**Table 3:** Themes Identified in the Post-Journey Interviews.

THEME 1: NEIGHBORHOOD CONTEXT	
Theme and Definition	Exemplary Quote
*Neighborhood Changes and Variation:* Direct or indirect transitions that citizen scientists indicate that have happened in their neighborhood- past, present, or predicted to happen in the future. Also includes citizen scientists’ perceptions of geographic, racial, and socioeconomic diversity within a neighborhood.	“…especially black families—they worked hard to get these homes…Um but there’s gentrification. So we have, you know, there’s new neighbors here including us so it’s interesting to see the dynamics come together.” “…forcing up the property value and thus the corresponding tax rates for a lot of indigenous residents… it’s actually making it harder to hold onto sort of the last relative affordable [housing] of the neighborhood in the city.” “…don’t have a lot of food options for people who live directly in this neighborhood. Magazine [Street] is a different world than the people who actually *live* in this neighborhood and what food purchasing they’re doing.” “Just how neighborhoods change in a very short amount of space is very different for me coming from the Midwest where you kind of have a progression generally from one socioeconomic neighborhood to another. Here it could be block to block.”
*Community Membership:* Perceived ability to call oneself a New Orleans “resident,” usually through the measure of time lived in their neighborhood or city. Long-time residents spoke with more confidence and ownership over their community’s needs and strengths. Whereas recent transplants provider disclaimers about how their perspective may or may not be representative of other residents’ experiences.	“I think like probably typical—I’m not from New Orleans—but probably typical to many New Orleans’ communities…” “Because I’ve been here so long, [laughs] I’ve been here so long.”
*Post-Katrina Sense of Place:* The ways in which Hurricane Katrina impacted citizen scientists’ perceptions around sense of place and neighborhood social cohesion.	“It is a story to tell especially after Katrina because 80% of the people that were here prior to Katrina is back and I think they deserve—well I’d like to see them have more and have a better standard of living that they had before Katrina.” “So after Katrina we moved to Houston… to really build a community sense beyond just neighbors—you don’t get that.”
*Neighborhood Cohesion and Pride:* One’s perceived connectedness of residents in the neighborhood and willingness to help each other. Also, one’s positive regard for their neighborhood.	“I really love my neighborhood” “…reinforce like the strengths that I see in the neighborhood. I do believe there is a strong neighborhood—if you decide to interact with your neighbors—like there’s a strong neighborhood aspect.”
THEME 2: REASONS FOR PARTICIPATING	
*Individual Level:* Individual level reasons include those that have to do with personal interest, health of oneself, or health of another individual.	“So this was just an opportunity to look at [my neighborhood] that way and capture it in a different way.” “This coincided with me discovering this vegan challenge…continue on with the lifestyle changes I am trying.”
*Community Level:* Community –level reasons include highlighting unseen neighborhood challenges, concern for sub-populations (i.e., youth), or hope for overall community health improvement.	“I feel like this part of like telling my story and other people telling their story would inform and better equip people to make decisions that could have lasting impact on people.” “I wanted to be able to be like a voice for my community for the positive and negative things that impact people’s access to resources contributing to their health behaviors.” “Because I guess everyone needs to know about the neighborhood. You know, there’s some good and there’s some bad in the neighborhood and there’s always room for improvement and hopefully this study will help improve this neighborhood.”
THEME 3: REALIZATIONS DURING THE STORYTELLING PROCESS	
*Realized Strengths/Positives:* Strengths and assets identified by citizen scientists through the storytelling process.	“That there are assets to this neighborhood that I forget about. Like that park is an asset that I don’t use as often…I should get out and walk.” “Oh wow! I didn’t know [Sprout NOLA Community Garden]!” “This didn’t really take that long and so it also sort of makes me think that I probably could make the effort just from a health perspective to get out of the office for 20–30 min a day”
*Realized Problems/Negatives:* Problems and needs identified by citizen scientists through the storytelling process.	“But it also reminds me of the need and just remembering the struggles that I have personally had in this neighborhood and that other people are probably still experiencing and so how to address that and how to help other people deal with their issues that they have in health and stuff.” “So things like going into corner stores and sharing things like the fried food and stuff…really puts me in the shoes of other people as well not just myself”
*Normalization of Health Barriers:* Issues that citizen scientists have become used to but identified as a health barrier through this storytelling process.	“Yeah I guess one thing is maybe the traffic on Claiborne [Avenue] and really noticing how much that influences things we do or we don’t do. Or how we do things.” “I guess how frequent um we do have broken sidewalks—I kind of know that, but I haven’t really walked the street or at least not in years since my children were with the bicycles…”
*Ability to Advocate:* One’s perceived ability and comfort with speaking up about issues in the neighborhood to people with the capacity to make change.	“We’re not the people who can change it. We can tell them the people but they’re like, ‘well nobody else is saying nothing’ or when we used to have community meetings and they would vent about stuff. But then when they would get in the meeting, they wouldn’t say anything!” “In order for there to be change you need to um advocate for that. And it’s your responsibility as an individual to communicate, um especially cus there’s a lot of people who don’t feel confident or capable of doing it…”
*Decisional Power:* Citizen scientists’ perceptions of who makes decisions and their ability to participate in and influence change at the community and policy level.	“I don’t know who to communicate with to make change—those channels aren’t clear.” “So really thinking about the people in the community and just hearing from them. And that story is going to help make changes that will last–because we can make a lot of changes but they don’t last.”

## Data Availability

The data presented in this study are available on request from the corresponding author. The data are not publicly available due to privacy; and that the study received IRB approval in 2018. Therefore, language around public data sharing was not included in the study consent form.
